# The Medical Society of the County of Erie

**Published:** 1892-08

**Authors:** Eli H. Long

**Affiliations:** Secretary


### Society Proceedings.

### THE MEDICAL SOCIETY OF THE COUNTY OF ERIE.

Reported by ELI H. LONG, M. D., Secretary.

### MORNING SESSION.

Semi-annual meeting held in the Y. M. C. A. Building, June 14, 1892. The President, Dr. W. W. Potter, called the meeting to order at 10 o'clock. The minutes of the annual meeting, held January 12, 1892, and of the two special meetings, held respectively February 20 and February 29, 1892, were read and adopted.

The committee on membership reported favorably upon the following-named candidates, who were duly elected members of the society, namely: Drs. W. J. Beutler, J. M. Hewitt, A. J. Colton, M. V. Ball, J. J. Drake, L. A. Denton, Mary J. Denton, H. U. Williams, J. T. Harris, E. J. Gilray, B. C. Johnson, A. E. Collins, and A. G. Bennett. The committee on membership further reported the receipt of applications for membership from the following named: Drs. Eleanor McAllister, Loren H. Staples, Carlos E. Bowman, Mary F. Greene, H. C. Leonhardt, Jane W. Carroll, Robert S. Hambleten, Frank J. Thornbury, Henry F. Carter, Charles H. Perry, and Alfred E. Diehl. These applications to lie over until the annual meeting in January, 1893, under the rule.

The special committee on the Practical Use of the Metric System, reported through its Chairman, A. L. Benedict, M. D., as follows:

### REPORT OF THE COMMITTEE TO INVESTIGATE THE PRACTICAL USE OF THE METRIC SYSTEM.

Acting in accordance with the directions received at the January meeting, your committee, appointed to investigate the workings of the metric system, soon issued a circular setting forth briefly the relations of the metric and apothecary's system. A return postal card was inclosed, asking replies to the following questions :

1. To what extent do you use the metric system ?

2. Do you favor the adoption of the metric system ?

3. What do you consider the principal obstacle to the adoption of the metric system ?

4. What suggestions have you to offer bearing on this subject ?

Although the aim has been to reach every member of the society, it is probable that not over 250 of the 270 members ever received the cards. Change of residence, loss in the mail, and consignment to the waste basket without reading, are reasons offered as explanations. Eighty-two, nearly a third of the total number of cards issued, have been returned filled out. The committee has been surprised and gratified both at the number of answers received and with the proportion who have expressed themselves as favoring the metric system. The eighty-two replies were assorted as follows :

1. From those who do not use the metric system at all, and who are opposed to its introduction—24.

2. Those who express themselves as indifferent as to whether the metric system is adopted or not—8.

3. Those who do not use the metric system, but who favor its adoption—16.

4. Those who use the metric system in a small proportion of their prescriptions, and who favor its adoption—17.

5. Those who use the metric system in at least fifty per cent. of their prescriptions, including a number whose use of the system is universal, or practically so—17.

The answers to the third question, What do you consider the principal obstacle to the adoption of the metric system ? may, with two or three exceptions, be summed up under the one word, *conservatism.* This conservatism, however, is looked at in different lights by different men. One who does not use the metric system at all, answers, " ignorance and laziness," to the question. Several quote such sayings as " Let well enough alone," or " 'Tis better to endure the evils which we have than fly to others which we know not of." Quite a few express the fear that the prevailing ignorance of the system among physicians and druggists would occasion serious mistakes. One gentleman, who has taken the pains to write an able and courteous letter on the subject, speaks of the danger which might arise during an epidemic, when an unusually large number of prescriptions would tax the capacity of the drug store. Several writers have favored the adoption if it could be done suddenly and universally. The gentleman just referred to, however, states that a law compelling the use of the metric system in England had to be repealed after a year's trial, on account of the blunders which occurred. The committee is quite in harmony with his further suggestion, and that of several other gentlemen, that no one should attempt to use the metric system until he is assured of his ability to do so correctly. As an illustration of the mistakes which may arise from a partial knowledge of the subject, might be mentioned a criticism from an anonymous correspondent. Comparing the apothecary's with the metric column of the prescription, used as an illustration in the circular, and seeing that ten (10) grams stands opposite to four (4) drachms, and overlooking the fact that the apothecary's prescription is for a twenty-four dose mixture, while the metric prescription is for a twenty dose mixture, and apparently overlooking, too, the explicit statement that the transposition is approximate so as to avoid awkward arithmetical expressions, he assumes an error on the part of one member of your committee. We are glad that this point has been brought up, for it calls attention to two distinct ways of using the metric system. In one the prescription is thought out in the apothecary's system, in the other the prescription is from the beginning genuinely metric. In transposing from one system to the other, the amount given at a single dose should first be calculated, and this quantity should then be translated into the nearest aliquot part of a gram, and the metric prescription should then be constructed independently from the single dose. In the particular case referred to, the four drachms of the tincture of the chloride of iron, etc., divided by twenty-four, gives an individual dose of ten minims. It is represented in the metric prescription by a fifty centigram dose, that is to say, very nearly eight minims. If the dose had been translated exactly, the metric prescription would have called for thirteen and a fraction grams.

One gentleman states that the principal obstacle to the adoption of the metric system is the fact that he has always used the other system. Quite a number ascribe the trouble to the lack of proper instruction in the metric system in medical colleges, and to the fact that in clinical lectures and journal articles the apothecary's system is usually employed. Many labor under what is certainly a misapprehension, that colleges of pharmacy do not prepare their graduates to dispense in the metric system. Two teachers in the University of Buffalo, and one in Niagara College, report that the medical students receive practical instructions in prescription writing in the metric system. Several replies contain the excellent suggestion that the metric system should be taught in public and private schools. Two or three gentlemen, curiously enough, oppose the metric system on the ground of its being French, forgetting the fact that the apothecary's system is not an American institution, nor even the special property of English-speaking nations. They also overlook the fact that the metric system, although originating with the French, has on its own merits so commended itself to Germany, Russia, Italy, Spain, Turkey, and, in fact, most foreign nations, that it is at present not a French but a cosmopolitan system.

Your committee represents, in its three members, various degrees of sentiment with regard to the metric system. It has endeavored to investigate the subject impartially, and to present in its report not so much its own opinions as the facts elicited by the replies to the circulars. About forty Buffalo physicians make, at least, partial use of the metric system, and about twenty-five[1] of these use it in fifty per cent., or more, of their prescriptions. Sixteen more physicians have been heard from, representing not only Buffalo but some of the smaller towns, who do not use the metric system, but who favor its adoption. The only objections offered to its use, in the replies to the circular, have been a disinclination to make a change and the danger of errors in compounding prescriptions. Among those who use the metric system are a number who write 2,000 or more prescriptions yearly, a physician whose general practice is probably as large as any in the city, two specialists who are acknowledged leaders in their respective branches, a man who has a large general and surgical practice, and who estimates his prescriptions at 6,000 annually. The committee would remind the members of the society that the metric system has been formally adopted in the United States Pharmacopeia, that Great Britain is the only nation of importance, besides the United States, that has not already put the metric system to a practical test and found it satisfactory; that, at least, two American wholesale drug houses

1. This number includes several who did not reply to the circular, and who are not counted in the table previously given.

have adopted it; that most of the other manufacturing chemists are paying some attention to it, especially in selecting for the amount of alkaloids contained in their pills such fractions of a grain as shall correspond to the milligram and its multiples; that the doses of most of the synthetic drugs, such as acetanilide, antipyrin, and the like, are established in those countries where the metric system is in use; that chemists and physicists do most of their calculating in the metric system; in short, that we are simply waiting for a current strong enough to loosen the anchor of conservatism from its hold on the rocks of habit and to bring our own barque into line with the vessels which carry the medical profession of other nations. Nothing succeeds like success, and nothing accomplishes so much as doing the work that lies before us. Let those who are prepared to use the metric system use it. Let those who feel that years of valuable and conscientious practice have established too firmly the habit of the older system, adhere to that. To clinical teachers, to editors of medical journals, and to writers of text-books, we look for such a double presentation of practical therapeutics as shall familiarize their hearers and readers with the metric system without detracting from the value which those accustomed to the apothecary's system may obtain from the subject.

On motion of Dr. W. W. POTTER (Dr. Stockton in the chair), the report was accepted and the committee continued, with instructions to further pursue its investigations and report at the annual meeting, in January, 1893.

The report of the Board of Censors was then called for, when Dr. EDWARD STORCK arose and stated that he had declined further to serve as chairman, but at the last annual meeting he had, nevertheless, been elected, and he now wished to present his resignation. Dr. Storck also gave notice of his intent to move at the next annual meeting that two members, who had previously been subjects of discipline by the society, be relieved from the censure resting upon them.

The SECRETARY presented the resignation of Dr. H. R. Hopkins as a member of the Board of Censors. The Chair directed that action be deferred on these resignations until the next annual meeting.

Under communications from abroad, the SECRETARY read a letter from the Medical Society of the State of New York, offering back volumes of the Transactions, and announcing the Merritt H.

Cash prize, which was open for competition by all members of county societies.

The scientific programme was then taken up, when the President, Dr. W. W. Potter, (Dr. A. A. Hubbell in the chair,) read a paper, entitled Asepsis and Antisepsis in the Lying-in-Chamber. This paper was discussed by Drs. J. C. Thompson, J. J. Walsh, W. C. Callanan, Joseph Price, L. S. McMurtry, Charles A. L. Reed, R. L. Banta, J. F. W. Ross, and the discussion was closed by Dr. Potter.

Dr. Chas. A. L. Reed, of Cincinnati, Secretary-General of the Pan-American Medical Congress, by invitation, then made some remarks on the development of the plan of the Congress, giving a synopsis of its history, and a *résumé* of its purposes.

The society then took a recess until 2.30 P. M.

AFTERNOON SESSION, 2.30 P. M.

The meeting was called to order promptly by the President, when Dr. James F. W. Ross, of Toronto, read a paper, entitled Actual, Not Text-Book, Experience with Cases of Ectopic Gestation. This paper was discussed by Drs. M. A. Crockett, W. S. Tremaine, F. W. Bartlett, Joseph Price, and in closing by the author.

The following group of papers was then read : Natural History of Pelvic Inflammation, by Joseph Price, M. D., of Philadelphia ; The Relation of So-called Minor Gynecological Operations to Pelvic Diseases, by L. S. McMurtry, M. D., Louisville, Ky. ; Methods of Early Diagnosis of Intra-Pelvic Diseases, by Charles A. L. Reed, M. D., of Cincinnati.

These papers were discussed in a group by Drs. Ingraham, Hayd, Hopkins, Price, Wells, McMurtry, and Reed.

Dr. H. R. Hopkins then offered the following :

Whereas, This society has had the rare privilege, at this meeting, of the presence of eminent and honored visitors and guests, viz. : Dr. James F. W. Ross, of Toronto ; Dr. Joseph Price, of Philadelphia ; Dr. Lewis S. McMurtry, of Louisville ; Dr. Charles A. L. Reed, of Cincinnati ; and Dr. Brooks H. Wells, of New York, and has also had the greater privilege and pleasure of listening to most profound and scholarly papers and practical and edifying discussions from these eminent visitors ; and,

Whereas, Professional courtesies of the kind just received by this society are only offered and received at the cost of great personal sacrifice of opportunity and energy ; therefore,

*Resolved,* That this, the Medical Society of the County of Erie, at its semi-annual meeting, June 14, 1892, does now and hereby tenders to Doctors Ross, Price, McMurtry, Reed, and Wells, a vote of thanks in testimony of its appreciation of their singular professional eminence and personal courtesy.

The preamble and resolution were adopted unanimously by rising vote.

The semi-annual meeting then, at 6.15 P. M., adjourned without day.

